# Acceptance of technology-enhanced learning for a theoretical radiological science course: a randomized controlled trial

**DOI:** 10.1186/1472-6920-12-18

**Published:** 2012-03-30

**Authors:** Emeka Nkenke, Elefterios Vairaktaris, Anne Bauersachs, Stephan Eitner, Alexander Budach, Christoph Knipfer, Florian Stelzle

**Affiliations:** 1 Department of Oral and Maxillofacial Surgery, Erlangen University Hospital, Erlangen, Germany; 2 Department of Oral and Maxillofacial Surgery, University of Athens Medical School, Attikon Hospital, Athens, Greece; 3 Department of Prosthodontics, Erlangen University Hospital, Erlangen, Germany

## Abstract

**Background:**

Technology-enhanced learning (TEL) gives a view to improved education. However, there is a need to clarify how TEL can be used effectively. The study compared students' attitudes and opinions towards a traditional face-to-face course on theoretical radiological science and a TEL course where students could combine face-to-face lectures and e-learning modules at their best convenience.

**Methods:**

42 third-year dental students were randomly assigned to the traditional face-to-face group and the TEL group. Both groups completed questionnaires before the beginning and after completion of the course on attitudes and opinions towards a traditional face-to-face lectures and technology-enhanced learning. After completion of the course both groups also filled in the validated German-language TRIL (Trierer Inventar zur Lehrevaluation) questionnaire for the evaluation of courses given at universities.

**Results:**

Both groups had a positive attitude towards e-learning that did not change over time. The TEL group attended significantly less face-to-face lectures than the traditional group. However, both groups stated that face-to-face lectures were the basis for education in a theoretical radiological science course.

The members of the TEL group rated e-mail reminders significantly more important when they filled in the questionnaire on attitudes and opinions towards a traditional face-to-face lectures and technology-enhanced learning for the second time after completion of the course.

The members of the technology-enhanced learning group were significantly less confident in passing the exam compared to the members of the traditional group. However, examination results did not differ significantly for traditional and the TEL group.

**Conclusions:**

It seems that technology-enhanced learning in a theoretical radiological science course has the potential to reduce the need for face-to-face lectures. At the same time examination results are not impaired. However, technology-enhanced learning cannot completely replace traditional face-to-face lectures, because students indicate that they consider traditional teaching as the basis of their education.

## Background

The growth in dental student numbers combined with limited availability of suitably trained teaching staff forces dental schools to reconsider their methods of teaching and learning [1]. Several aspects of dentistry are performed with the use of information technology (IT). IT is also increasingly prevalent in dental education. It has been hypothesized that the technological shift may help dental education to meet expectations for higher-quality education while at the same time funds are reduced [2-4]. It has been said that e-learning offers numerous advantages for the learner. Especially it increases the student's independence by an "anytime, anywhere" access to educational opportunities [5-7]. With technological advances comes the challenge of how best to use them in dental education. Educational researchers are challenged to test the effectiveness and efficiencies of these new methods of online learning to try to demonstrate their ability to be as effective as traditional methods of teaching [8]. Technology acceptance research is considered a mature field in information systems research, with many models and theories developed and tested[9]. However, despite the large volume of work in this area, only limited research has been conducted in the health care context [10]. Nevertheless, in recent years, more and more dental schools have started to support face-to-face teaching by online platforms [11-15]. While the learning process can be richly supported using e-learning technologies, learning is still intrinsically a cognitive and embodied phenomenon. Therefore, the term technology-enhanced learning may better represent the relationships between technology and learner [16]. It has been shown that technology-enhanced learning can be as effective as traditional formats [17]. However, it cannot be concluded that technology-enhanced learning is more popular than traditional forms of medical education [18]. Therefore, there is a clear need to clarify when to use technology-enhanced learning and how to use it effectively [19].

The present study compared a traditional face-to-face course on theoretical radiological science with a technology-enhanced learning course where students could combine face-to-face lectures and online modules at their best convenience. The study aimed at assessing

i) students' attitudes and opinions towards the different alternatives in teaching and learning and

ii) examination results that the different approaches yielded

## Methods

The study was approved by the institutional Ethics Committee of the University of Erlangen-Nuremberg. 42 third-year dental students were included in the study. They were scheduled for the theoretical radiological science course. The learning content comprised radiation physics, X-ray production, X-ray interactions, radiation dose, imaging equipment, radiation protection, image creation, and normal radiological anatomy of the teeth and jaws.

In an introductory explanatory face-to-face session the conventional face-to-face theoretical radiological science course and the technology-enhanced learning course were introduced to the students. Participation in the study was optional. Subsequently, the students were randomly assigned to two groups and their demographic data were documented. Each participant was asked to open an envelope containing his or her group allocation. Block randomization and sequentially numbered sealed opaque envelopes were used for the allocation[20].

One group followed the traditional face-to-face course (traditional group). For the second group the e-learning modules were available on the Internet (technology-enhanced learning group). The content of the technology-enhanced learning course was equivalent to that of the traditional course. For the students of the technology-enhanced learning group the attendance of the face-to-face lectures was optional. It was explained to them that they could combine the technology-enhanced learning course with the face-to-face lectures at their best convenience.

The students of the technology-enhanced learning group received information on how to log-on to the online material that was available on the Internet. The online platform was provided by the Virtual University of Bavaria (Virtuelle Hochschule Bayern, http://www.vhb.org). The students were asked to document the time they spent using the online material. At the end of the introductory session another optional session was offered to the members of the technology-enhanced learning group to get familiar with the sign-in procedure to the e-learning platform and its use in order to avoid non-users of the e-learning platform in this group.

During the introductory session all students of both groups had to complete anonymously a questionnaire on their attitude towards face-to-face lectures and e-learning. The questionnaire had been put together by the authors. A pilot version of the questionnaire had been tested before by 20 fifths-year dental students who had experience with technology-enhanced learning. Based on their input some questions were rephrased, some questions were deleted and a number of additional questions was added. The 30 questions of the final version focused on eliciting students' prior e-learning experience, and attitudes and perceptions regarding e-learning and face-to-face lectures. The answers could be given on a scale from 1 to 6 (1 = I totally disagree, 6 = I totally agree, Appendix 1).

The traditional course was delivered as eight didactic lectures of 45 minutes each over a period of two months. The PowerPoint presentation of each lecture was available for downloading on an online platform named Medlearn http://http//medlearn.uni-erlangen.de before each lecture was held. This online platform is provided by the Erlangen Medical and Dental School. Attendance of the face-to-face lectures was documented for each student of the traditional group as well as for each student of the technology-enhanced learning group.

The e-learning content for the technology-enhanced learning group comprised eight online modules. They included texts, images and graphs, videos, hyperlinks, reference lists with recommended supplementary literature and tests. Providing the learning objects in many different ways and by the use of different media aimed at making e-learning an active and constructive process that was self-directed by the student [21]. The online modules could be accessed via the Internet with any personal computer. However, it was mandatory to sign in to the e-learning platform in order to get access to the online content. The e-learning modules were constantly available to be accessed wherever and whenever the students chose up until the assessment examination. The number of students of the technology-enhanced learning group who signed in to the e-learning platform, was documented.

Each of the eight online modules was planned to be worked through within one week. At the beginning of each week the students of the technology-enhanced learning group were invited via e-mail to start working on a specific module. Each module started with a short outline of the specific learning targets and the time needed to go through the material. A short summary of the most important aspects of the module was included. A number of initial multiple choice questions gave the students the opportunity to check in how far they were already familiar with the content of the modules. A reminder to work through the module was sent after three days.

The learning content of the online modules was detailed in written text that included "didactical boxes". These didactical boxes highlight the most important aspects of the learning content. At the end of each module again a number multiple choice questions were given to allow checking the knowledge acquired. The e-learning system provided feedback to the students which answers were given correctly and indicated the right answers for questions that were not answered correctly.

The Medlearn online platform allowed posting questions concerning the learning content and other general aspects. All questions were answered within 24 hours by the academic staff. All questions that were asked via the Medlearn online platform were documented. Also all questions that were asked during the face-to-face lectures were documented. One week after the end of the theoretical radiological science course the students of both groups were asked to fill in the questionnaire on their attitude towards face-to-face lectures again together with the TRIL questionnaire which is a validated modular German-language questionnaire (TRIL, Trierer Inventar zur Lehrevaluation) for the evaluation of courses at university (Appendix 2) [22]. It comprises 6 topics. Topic 1 ("structure and didactics") consists of 7 questions that concerned the lecturer's skills in didactics and structuring of the learning content. Topic 2 deals with the motivational skills of the lecturer consisting of 5 questions. Topic 3 (4 questions) addresses the lecturer's skills in creating a favorable climate during the course. Topic 4 consisted of 3 questions and asks the students to evaluate practical relevance of the course by providing a connection between theory and practice. Topic 5 subsumes 5 questions on different additional aspects of courses. Topic 6 consisted out of 8 questions that concerned the e-learning material. This topic had only to be answered by the students who used the technology-enhanced learning course.

Two weeks after the end of the theoretical radiological science course a multiple choice exam was held. 20 questions had to be answered. The students had to answer 12 questions correctly to pass the exam.

### Statistical analysis

Mean values are given with standard deviations. For comparison of continuous variables in paired samples, the Wilcoxon test was used, while for unpaired samples the Mann-Whitney-U test was adopted. The χ^2 ^test was used to test if there was a statistically significant difference in the gender distribution between the two groups. Spearman's rho was calculated in order to check if there was a correlation between the time spent using the e-learning modules and the results of the final examination. P-values less than or equal to .05 were considered significant. All calculations were made using SPSS Version 14.0 for Windows (SPSS, Chicago, USA).

## Results

All 42 students chose to join the study. There was no statistically significant difference in age between the traditional group and the technology-enhanced learning group (24.3 ± 2.7 years in the traditional group, 24.2 ± 2.5 years in the technology-enhanced learning group, p = .990). In both groups there were more females than males (12 females and 9 males in the traditional group, 14 females and 7 males in the technology-enhanced learning group). However, the distribution in gender did not differ statistically significantly between the two groups (p = .525).

During the scheduled period of the theoretical radiological science course no technical problems were encountered with the online platforms. All face-to-face lectures took place as scheduled. All questionnaires that were filled in before and after the theoretical radiological science course were adequately completed and returned.

The additional session for the members of the technology-enhanced learning group to get familiar with the e-learning platform was attended by 6 female students. Consequently, all 21 members of the technology-enhanced learning group logged on to the e-learning platform. There were no non-users of the e-learning platform in the technology-enhanced learning group.

The answers to the questionnaire concerning attitudes and opinions towards face-to-face lectures and e-learning filled in during the introductory session did not show statistically significant differences between the traditional group and the technology-enhanced learning group (Table 1). The students of both groups indicated that they already had experience with the use of online learning material (Question 1, Table 1). Both groups were positive about the flexibility that e-learning can give as far as time and place of learning are concerned (Question 16 and Question 17, Table 1). Both groups also were convinced that they could pass the exams after the e-learning course as successfully as they would have been after attending the face-to-face lectures (Question 21, Table 1). However, the students of both groups indicated that they would not appreciate more e-learning courses during their studies at university (Question 22, Table 1). The answers of both groups indicated that face-to-face lectures were still considered the basis for learning at university and that the lecturer had a strong influence on the students' interest in a specific subject (Question 5 and Question 8, Table 1). The answers to the Questions 13, 14 and 23 showed that both groups preferred to combine face-to-face lectures and e-learning courses with the supplementary use of books and other electronic media (Table 1).

**Table 1:** Data of the answers to the questionnaire on students' attitudes and opinions towards face-to-face lectures and e-learning before the beginning and after the completion of the theoretical radiological science course.

Question **No**.	Questionnaires filled in before the beginning of the course							Questionnaires filled in after completion of the course						
	
	Traditional group			Technology-enhanced learning group			p	Traditional group			Technology-enhanced learning group			p
				
	n	Mean value	SD	n	Mean value	SD		n	Mean value	SD	n	Mean value	SD	
1	21	4.6	1.8	21	4.7	1.3	.937	21	4.5	1.8	21	3.9	1.7	.206

2	21	3.9	1.6	21	3.2	1.9	.196	21	4.5	1.7	21	3.9	1.8	.241

3	21	4.9	1.4	21	4.3	1.6	.240	21	4.6	1.5	21	4.8	1.5	.537

4	21	4.6	1.5	21	4.4	1.4	.639	21	5.1	1.1	21	4.8	1.5	.679

5	21	3.9	1.4	21	4.0	1.4	.846	21	4.1	1.5	21	4.1	1.6	918
6	21	3.9	1.4	21	3.8	1.2	.979	21	3.4	1.7	21	3.3	1.8	.858

7	21	5.7	.6	21	5.7	.9	.738	21	5.9	.3	21	5.7	.9	.607

8	21	4.9	.7	21	4.9	1.1	.577	21	5.0	.9	21	5.2	.9	.391

9	21	3.9	1.5	21	3.4	1.6	.349	21	3.5	1.4	21	3.4	1.4	.606

10	21	4.7	1.1	21	4.4	1.3	.372	21	4.7	1.1	21	4.7	1.1	1

11	21	4.3	1.4	21	3.9	1.5	.367	21	4.3	1.5	21	4.0	1.2	.380

12	21	4.9	1.3	21	4.4	1.4	.196	21	4.7	1.5	21	4.0	1.7	.128

13	21	5.1	1.0	21	4.9	1.1	.507	21	5.1	1.0	21	4.9	1.1	.532

14	21	3.5	1.4	21	3.7	1.3	.643	21	3.8	1.3	21	3.9	1.0	.896

15	21	3.2	1.2	21	3.0	1.0	.516	21	3.1	1.3	21	3.1	1.4	.918

16	21	4.2	1.7	21	3.8	1.6	.337	21	3.8	1.7	21	3.7	1.9	.980

17	21	4.4	1.2	21	4.1	1.6	.607	21	3.6	1.7	21	3.8	2.0	.635

18	21	5.2	.9	21	5.4	1.2	.354	21	5.6	.7	21	5.5	1.0	.817

19	21	4.5	1.2	21	4.4	1.1	.805	21	4.5	1.1	21	3.7	1.7	.135

20	21	2.9	1.5	21	2.9	1.2	.837	21	3.5	1.7	21	3.4	1.6	.858

21	21	4.4	1.0	21	3.6	1.4	.058	21	4.5	1.4	21	3.3	1.7	.020

22	21	2.9	1.4	21	2.9	1.3	.969	21	2.6	1.5	21	3.0	1.8	.570

23	21	5.2	1.0	21	4.9	1.1	.453	21	4.7	1.6	21	4.5	1.5	.402

24	21	4.2	1.0	21	4.4	1.5	.264	21	4.6	1.3	21	3.9	1.7	.181

25	21	5.1	1.3	21	4.8	1.3	.248	21	5.3	.8	21	4.4	1.6	.074

26	21	4.9	1.3	21	4.2	1.6	.125	21	4.3	1.3	21	4.1	1.5	.678

27	21	4.9	1.1	21	4.7	1.3	.664	21	4.8	.9	21	4.3	1.5	.373

28	21	3.5	1.1	21	3.3	1.3	.517	21	3.2	1.4	21	3.0	1.2	.650

29	21	4.4	1.3	21	3.9	1.6	.214	21	4.5	1.5	21	5.0	1.1	.060

30	21	5.1	.8	21	4.8	1.1	.382	21	4.8	1.2	21	4.3	1.7	.558

Answers could be chosen between 1 and 6 (1 = I totally disagree, 6 = I totally agree)

The complete questionnaire is given in Appendix I

All members of the traditional group followed all 8 lectures. 11 members of the technology-enhanced learning group attended the first face-to-face lecture. The final lecture of the theoretical radiological science course was only attended by 4 of 21 members of the technology-enhanced learning group. The technology-enhanced learning group attended the face-to-face lectures significantly less than the traditional group (8 ± 0 lectures attended by the traditional group, 2.5 ± 3.2 lectures attended by the technology-enhanced learning group, p < .0005).

During the theoretical radiological science course no questions were posted on the online platform. During the face-to-face lectures all questions that were asked concerned the congruence of the learning content given in face-to-face lectures and the content provided in the online modules. All questions were asked by members of the technology-enhanced learning group.

In the technology-enhanced group 6 students used the e-learning modules up to 60 minutes, 4 students 421 to 480 minutes, 4 students 481 to 540 minutes, 5 students 541 to 600 minutes and 2 students over 600 minutes.

When the students filled in the questionnaire concerning their attitudes towards face-to-face lectures and e-learning after the completion of the theoretical radiological science course there was a statistically significant difference between the traditional group and the technology-enhanced learning group for Question 21 (4.5 ± 1.4 for the traditional group and 3.3 ± 1.7 for the technology-enhanced learning group, p = .020, Table 1). The answers to this question revealed that the members of the technology-enhanced learning group were less confident in being successful in the exam compared to the traditional group. Moreover, the technology-enhanced learning group rated e-mail reminders significantly more important than the first time they filled in the questionnaire (Question 29, 3.9 ± 1.6 before the start of the course and 5.0 ± 1.1 after completion of the course, p = .025).

The answers to the TRIL questionnaire revealed that the students of both groups rated the structure and the didactics of the course positively (Table 2). The ratings for topic 2 showed that the students were satisfied with the lecturer's ability to explain difficult learning content in an understandable way (Question 8, Table 2). There was no statistically significant difference in the ability to keep their concentration during the course between the students of the two groups (Question 11, Table 2). The students of both groups appreciated the good climate during the course when giving the ratings for topic 3. In topic 4 the students of both groups clearly could identify a connection between the theoretical radiological science course and the practical relevance of the learning content (Table 2). For topic 5 the students of both groups indicated that the degree of difficulty of the courses was adequate (Question 23, Table 2). The members of both groups stated that they were interested in the theoretical radiological science course (Question 24, Table 2).

**Table 2:** Results of the answers to the TRIL (Trierer Inventar zur Lehrevaluation) questionnaire filled in by the students after the completion of the theoretical radiological science course.

**Question No**.	Traditional group			Technology-enhanced learning group			p
		
	n	Mean value	SD	n	Mean value	SD	
Topic 1 Structure and didactics							

1	21	4.1	1.3	21	3.9	1.1	.493

2	21	3.9	1.0	21	3.9	1.0	.824

3	21	4.3	1.1	21	3.7	1.1	.107

4	21	4.2	1.2	21	3.7	1.1	.201

5	21	5.0	.7	21	4.5	1.0	.122

6	21	4.8	1.0	21	4.5	.9	.304

7	21	4.7	1.2	21	4.5	1.0	.504

Topic 2 Motivational skills of the lecturer							

8	21	3.9	1.2	21	3.8	.8	.684

9	21	4.8	1.0	21	4.4	1.1	.367

10	21	3.9	1.5	21	3.4	1.1	.144

11	21	3.6	1.6	21	2.9	1.4	.215

12	21	3.1	1.6		2.8	1.2	.588

Topic 3 The lecturer's skills in creating a favorable climate							

13	21	5.1	1.0	21	4.7	1.2	.255

14	21	5.3	1.0	21	5.4	.7	.875

15	21	5.0	.9	21	5.3	.8	.426

16	21	5.2	1.0	21	4.9	1.0	.208

Topic 4 Practical relevance of the course							

17	21	4.4	.9	21	4.1	1.0	.240

18	21	4.6	.8	21	4.0	1.1	.077

19	21	3.5	1.1	21	3.4	1.2	.686

Topic 5 Questions of different additional aspects							

20*	21	1.8	.4	21	1.7	.5	.116

21	21	2.4	1.0	21	2.6	.9	.570

22	21	2.4	1.2	21	2.6	1.1	.304

23**	21	3.2	.4	21	3.3	.4	.435

24	21	3.8	1.0	21	3.8	1.0	.624

Topic 6 E-learning							

25	/	/	/	21	4.3	1.3	/

26	/	/	/	21	5.1	1.1	/

27	/	/	/	21	1.9	1.1	/

28	/	/	/	21	3.8	.9	/

29	/	/	/	21	4.7	.6	/

30	/	/	/	21	2.2	.7	/

31	/	/	/	21	1.7	.8	/

32	/	/	/	21	4.7	1.0	/

The complete questionnaire is given in Appendix II

Answers could be chosen between 1 and 6 (1 = I totally disagree, 6 = I totally agree)

*Question 20 could only be answered with 1 = yes or 2 = no. For the statistical analysis of these data the χ^2 ^test was used

**For Question 23 possible answers were 1 = too low, 2 = low, 3 = adequate, 4 = high and 5 = too high

Answers to topic 6 "e-learning" were only given by the members of the technology-enhanced learning group. These students stated that the all the necessary material was provided on the e-learning platform (Question 25, Table 2). They found that the material was easy to use (Question 26, Table 2) and facilitated gaining knowledge on the topic (Question 28, Table 2) instead of making learning complicated (Question 27, Table 2). The introduction to the e-learning course was rated clearly understandable (Question 29, Table 2). The students denied that the e-learning material was not necessary for the course (Question 30, Table 2). They stated that it was easy to orientate within the e-learning platform (Question 32, Table 2) and that the e-learning material was not assembled in a confusing way (Question 31, Table 2).

The examination results showed that there was no statistically significant difference between the traditional group and the technology-enhanced learning group (18.6 ± 1.2 points in the traditional group, 18.3 ± 1.3 points in the technology-enhanced learning group, p = .449). For the members of the technology-enhanced learning group there was no statistically significant correlation between the number of lectures attended and the examination results (Spearman's rho = .107, p = .654). The same was true for the time the technology-enhanced learning group spent in using the e-learning modules (Spearman's rho = .278, p = .071).

## Discussion

With the amount of medical information doubling nearly every seven years, it is obvious that education will be increasingly dependent on information technology to enable teachers and learners to cope with the growing amount of information necessary to keep up-to-date in their field [23,24]. E-learning is frequently used in many aspects of medical training [25]. Without doubt it has gained its place in medical education [26]. However, there is still not enough knowledge on how it can be used in the most effective way to enhance teaching and learning. The present study compared a traditional face-to-face course on theoretical radiological science with a technology-enhanced learning course where students could combine face-to-face lectures and online modules at their best convenience. The study aimed at assessing students' attitudes and opinions towards the different alternatives in teaching and learning and the examination results that the different approaches yielded.

An important role has been attributed to e-learning because of the rapid increase of biomedical knowledge [18]. E-learning is flexible and can deal with the increasing amounts of information taught in medical curricula [27]. However, it has been found previously that technology-dependent forms of medical education are less heavily used and are not thought to be as useful as traditional forms [18]. Also in the present study students considered face-to-face lectures the basis for education at university, although they rated the e-learning course positively.

International standards (for example ISO 9126) provide evaluation metrics that assume that the end user's acceptance of a technology in question is entirely dependent on software or hardware. However, this is only the case when there is external pressure or internal impetus for the application of software or hardware [28]. Sometimes the advantages of a technology are less obvious to a user. For instance in the present study the students of the TEL group were free to use or not to use technology-enhanced learning. In such cases, the software must also offer a number of other attributes. These must include a guarantee of safety, motivation and dependability [28]. The latter is not just about the hardware and software operating to specification, but is also a reflection of how well the technical system fits into the environment where it is used [29]. It seems that future research activities should focus on these aspects in order to significantly increase the acceptance of technology-enhanced learning. In the past information technology acceptance research has yielded many competing models. For an overview see [9]. The Technology Acceptance Model (TAM) has been designed to explain and predict user acceptance [30]. The model suggests that when users are confronted with a new software, certain factors influence their decision about how and when they will use the software [31]. The most important factors are perceived usefulness (the degree to which a person believes that using a particular system would enhance his or her job performance) and perceived ease-of-use (the degree to which a person believes that using a particular system would be free from effort). However, some authors have stated that technology acceptance models do not fit well with healthcare applications [32]. In such a context, users focus on usefulness. The perceived ease of use is not considered critical. In the present study, the members of the technology-enhanced learning group put the usefulness of the system into question. They were significantly less confident in being successful in the exam when compared to the traditional group. This aspect maybe is related to a criticism sometimes made of e-learning. Often e-learning is perceived as remote and impersonal [33]. However, learning has been identified being a social process. Interaction between learners and between learners and teachers can enhance the participation in learning activities [34]. Interestingly, online platforms for discussion in courses do not seem to fulfill this need in an adequate way, because students do not tend to use them in a number of different e-learning scenarios [35]. In the present study, the members of the technology-enhanced learning group had the clear advantage over the traditional learning group that they could contact the academic staff online all the time, if there were questions. However, they did not make use of this possibility.

The need for direct interaction is one of the major reasons for the fact that face-to-face lectures will always have their place in medical education. Today, the key point is to support the face-to-face activity effectively by e-learning. In the present study the support by e-learning significantly reduced the need for face-to-face lectures. The scenario of the present study gives a view to future developments where face-to-face lectures might be given on demand in a more flexible way whenever students feel the need to back up their e-learning activities with a traditional direct contact to the teacher. In this context it should be made clear that the present generation of learners does not see e-learning as a separate entity or special activity [26]. Each learner has his own approach to learning. He will adapt to his own personal circumstances, whether it is his preferred style of learning, the use of a favorite technology or to fit in with the competing pressures within his life. It has to be accepted as a simple fact that not all learners will use all of the e-learning resources all the time for their learning [26]. As a consequence, the use of technology-enhanced learning as adopted in the present study gives a good view to allowing every individual student choosing relevant bits from e-learning and traditional face-to-face lectures and combining them to his best convenience. One should not forget that e-learning is not always the best learning strategy for education [27]. This aspect has been taken into account in the present study. The students were enabled to opt for e-learning when it fits well within their learning demands. Additional future research will allow increasing the depth understanding of using technology to enhance teaching and learning effectively [16]. In this context, a lack of computer knowledge and skills has been identified as a major barrier in adopting e-learning in the practice of learning [34]. This aspect shows gender differences that lead to a reduced acceptance of e-learning in female students [36]. This problem was avoided in the present study by offering an additional optional introductory session to e-learning. This additional session was exclusively used by female students. As a consequence, there were no female e-learning non-users in the present study.

A number of other practical aspects of e-learning have been discussed in the past. For example e-mail reminders have been used effectively in the past to increase the use of corresponding e-learning systems [17]. The probability to logon to a specific e-learning system was increased by factor 8 when these reminders were sent to the participants. Therefore, it was decided to also use e-mail reminders in the present study.

The technology-enhanced learning group attended significantly less face-to-face lectures compared to the traditional group. However, the results of the exams of the technology-enhanced learning group showed no statistically significant differences compared with that of the traditional face-to-face group. The reduced attendance of face-to-face lectures took place without worsening the examination results. Therefore, it seems that the scenario of technology-enhanced learning increases the flexibility of the students as far as the attendance of face-to-face lectures is concerned without increasing the risk of poor examination results. Although the students of the technology-enhanced learning group feared to be less successful in the examinations than the traditional group, the reality proved them wrong. Compared to blended-learning concepts, where e-learning and face-to-face phases have a strict schedule, the technology-enhanced scenario described in the present study might be more convenient to the students while yielding acceptable examination results [35].

It can be assumed that not all e-learning approaches are equally effective and that variations in instructional design can influence learning outcomes. In this context the effectiveness of self-assessment questions has been stressed [37]. In the e-learning modules of the present study such self-assessment questions were available at the beginning and at the end of each module. However, the possibility of doing self-assessment did not lead to examination results that were superior to that gained by the conventional face-to-face lectures without self-assessment.

## Conclusions

The results of the final examinations revealed that students using technology-enhanced learning performed comparable to their counterparts who attended traditional face-to-face lectures. Although students of both groups rated e-learning positively, they stated that they still considered face-to-face lectures the basis of education at university. It seems that technology-enhanced learning in a theoretical radiological science course has the potential to reduce the need for face-to-face lectures. However, it cannot replace traditional education. Instead, it allows combining e-learning and face-to-face lectures at the best convenience of the students.

## Competing interests

The authors declare that they have no competing interests.

## Authors' contributions

EN led on conception, design, statistical analysis and interpretation of the data. EV contributed to drafting and revising of the article and critically appraising the content. SE contributed to drafting and revising of the article and critically appraising the content. AB was responsible for design and execution of the study. CK contributed to execution of the study and drafting of the manuscript. FS led on conception, design, statistical analysis and interpretation of the data. All authors have approved the final version of the article submitted.

## Appendix A. Appendix 1

Questionnaire on students' attitudes and opinions towards face-to-face lectures and e-learning. Answers to the questions could be chosen between 1 and 6 (1 = I totally disagree, 6 = I totally agree).

Q1. I have already made experiences with online material at university.

Q2. It is important for me to have the possibility to ask questions to the lecturer during face-to-face lectures.

Q3. I like to post questions to the lecturer on an online platform independent of the face-to-face lectures.

Q4. A face-to-face course where attendance is not mandatory, makes it easier for me to manage my studies at university.

Q5. Face-to-face lectures are most important for me to gain knowledge.

Q6. Gaining knowledge using an e-learning course is an important alternative to face-to-face lectures for me.

Q7. It is important for me to have the PowerPoint presentation at hand before the lecture takes place.

Q8. The lecturer has an important influence on my interest in a specific subject.

Q9. It is important for me to know before each face-to-face lecture what the learning targets are and which topics are lectured to prepare myself for the lecture.

Q10. A face-to-face lecture on a regular basis is helpful to me to get an overview on the learning matter and to structure learning.

Q11. I am able to organize my learning independent of the kind of knowledge transfer (face-to-face lecture, e-learning).

Q12. In order to extend my knowledge in a specific subject I use electronic media on a regular basis.

Q13. Besides face-to-face lectures books are the most important possibility for me to gain knowledge.

Q14. Besides face-to-face lectures electronic media are the most important possibility for me to gain knowledge.

Q15. E-learning supports me in attuning my learning to the several courses at university.

Q16. The flexibility in locality that e-learning provides, for me is a major advantage compared to face-to-face lectures.

Q17. The flexibility that e-learning provides as far as time is concerned, for me is a major advantage compared to face-to-face lectures.

Q18. When I attend an e-learning course which is the alternative to a face-to-face course on the same subject, nevertheless I'd like to have access to the PowerPoint presentations of the face-to-face course.

Q19. When I attend an e-learning course, I also join the face-to-face lectures on the same subject.

Q20. When an e-learning course and a face-to-face course are given on the same subject and are containing the same learning content, I will no longer join the face-to-face lectures.

Q21. I am able to gain an examination result by just joining an e-learning course that is as good as what I would have achieved by attending a face-to-face course.

Q22. I would like to have more e-learning courses at university.

Q23. When I attend an e-learning course, I will still use books for exam preparation.

Q24. Direct contact to the lecturer during face-to-face lectures facilitates preparation for the exams compared to just using the material of the e-learning course.

Q25. If there are aspects that I do not understand by using the e-learning material, I choose the possibility to ask my questions to the lecturer during a face-to-face lecture.

Q26. If there are aspects that I do not understand by using the e-learning material, I ask my questions to the lecturer by e-mail or post them on an online-platform.

Q27. If other students ask questions to the lecturer by e-mail or post questions on an online platform and answers are given, I use this material to check my knowledge.

Q28. I find it acceptable to gain knowledge on my own by working through an e-learning course.

Q29. It is a good support for me being reminded on the weekly e-learning module I have to work through by e-mail.

Q30. Having the possibility to choose between e-learning and face-to-face lectures makes it easier for me to manage my studies at university.

## Appendix B. Appendix 2

TRIL (Trierer Inventar zur Lehrevaluation) questionnaire for the evaluation of courses at university. Answers to the questions could be chosen between 1 and 6 (1 = I totally disagree, 6 = I totally agree) except for Questions 20 and 23.

*Question 20 could only be answered with 1 = yes or 2 = no. For the statistical analysis of these data the χ2 test was used.

**For Question 23 possible answers were 1 = too low, 2 = low, 3 = adequate, 4 = high and 5 = too high.

Topic 1 "structure and didactics"

Q1. The materials (manuscripts, PowerPoint slides, etc.) provided during the course were helpful for the understanding of the learning content.

Q2. The learning content was adjusted to the state of knowledge of the students.

Q3. Didactical materials (flipchart, PowerPoint slides, etc.) were used in an adequate way.

Q4. The lecturer gave short summaries in order to make clear which were the crucial points for the understanding of the topic.

Q5. The time management of the lecturer was adequate.

Q6. The learning content of the single sessions was adjusted to the learning targets.

Q7. The course had a reproducible structure.

Topic 2 "motivational skills of the lecturer"

Q8. The lecturer was able to explain difficult learning content in an understandable way.

Q9. The lecturer as able to keep contact to the audience (e.g. by eye-contact).

Q10. The lecturer created an inspiring atmosphere.

Q11. It was easy for me to remain concentrated during the course.

Q12. I was inspired to follow the train of thoughts during the course.

Topic 3 "the lecturer's skills in creating a favorable climate"

Q13. The style of speech of the lecturer was fluently and clear.

Q14. The lecturer treated the students friendly and was open-minded.

Q15. The lecturer allowed asking questions that concerned the learning content and answered them adequately.

Q16. The lecturer was able to be responsive to suggestions of the students concerning structure and organization of the course.

Topic 4 "practical relevance of the course"

Q17. During the course the relation between theoretical knowledge and practical application demonstrated.

Q18. The learning content of the course was adequately illustrated by practical examples (case studies, clinical applications, etc.).

Q19. I was inspired to deal with the learning content critically.

Topic 5 "questions on different additional aspects"

Q20*. I have contacted the lecturer at other occasions than the lecture.

1 = yes, 2 = no.

Q21. I prepared myself for the lectures on a regular basis (e.g. by reading of additional literature).

Q22. I did follow-up course work on a regular basis (e.g. by discussion with other students or by reading of additional literature).

Q23**. The degree of difficulty of the course was 1 = too low, 2 = low, 3 = adequate, 4 = high, 5 = too high.

Q24. I was interested in the course.

Topic 6 "e-learning"

Q25. All the materials necessary to follow the course has been provided online.

Q26. The e-learning material could be accessed without problems.

Q27. The e-learning material made learning more complicated.

Q28. The e-learning material facilitated learning.

Q29. There was a proper instruction for the use of the e-learning tool.

Q30. The e-learning material was not necessary for the successful attendance of the course.

Q31. The e-learning material was assembled in a confusing way.

Q32. It was easy for me to orientate myself in the e-learning material.

## Pre-publication history

The pre-publication history for this paper can be accessed here:

http://www.biomedcentral.com/1472-6920/12/18/prepub
